# An unusual inherited electroretinogram feature with an exaggerated negative component in dogs

**DOI:** 10.1111/vop.12998

**Published:** 2022-06-17

**Authors:** Simon M. Petersen‐Jones, Nate Pasmanter, Laurence M. Occelli, Kristen J. Gervais, Freya M. Mowat, Janice Querubin, Paige A. Winkler

**Affiliations:** ^1^ Department of Small Animal Clinical Sciences College of Veterinary Medicine, Michigan State University East Lansing Michigan USA; ^2^ South Shore Animal Hospital Boston Massachusetts USA; ^3^ Department of Surgical Sciences School of Veterinary Medicine, and Department of Ophthalmology and Visual Sciences School of Medicine and Public Health University of Wisconsin‐Madison Madison Wisconsin USA

**Keywords:** canine, electroretinography, photopic negative response, scotopic threshold response, SD‐OCT

## Abstract

**Objectives:**

To assess an inherited abnormal negative response electroretinogram (NRE) that originated in a family of Papillon dogs.

**Animals Studied:**

Thirty‐eight dogs (Papillons, or Papillon cross Beagles or Beagles).

**Procedures:**

Dogs underwent routine ophthalmic examination and a detailed dark‐adapted, light‐adapted and On–Off electroretinographic study. Vision was assessed using a four‐choice exit device. Spectral‐domain optical coherence tomography (SD‐OCT) was performed on a subset of dogs. Two affected males were outcrossed to investigate the mode of inheritance of the phenotype.

**Results:**

The affected dogs had an increased underlying negative component to the ERG. This was most pronounced in the light‐adapted ERG, resulting in a reduced b‐wave and an exaggerated photopic negative response (PhNR). Changes were more pronounced with stronger flashes. Similarly, the On‐response of the On–Off ERG had a reduced b‐wave and a large post‐b‐wave negative component. The dark‐adapted ERG had a significant increase in the scotopic threshold response (STR) and a significant reduction in the b:a‐wave ratio. Significant changes could be detected at 2 months of age but became more pronounced with age. Vision testing using a four‐choice device showed affected dogs had reduced visual performance under the brightest light condition. There was no evidence of a degenerative process in the affected dogs up to 8.5 years of age. Test breeding results suggested the NRE phenotype had an autosomal dominant mode of inheritance.

**Conclusions:**

We describe an inherited ERG phenotype in Papillon dogs characterized by an underlying negative component affecting both dark‐ and light‐adapted ERG responses.

## 1 INTRODUCTION

The electroretinogram (ERG) is a useful tool for assessing retinal function. It is widely used in veterinary ophthalmology for detection of retinal dysfunction such as that resulting from inherited retinal dystrophies and conditions such as sudden acquired retinal degeneration. It is also commonly used to screen eyes for normal retinal function prior to cataract surgery. In the research setting, it is used to further understand retinal function, investigate functional changes resulting from different retinal dystrophies and to assess responses to therapies.^1^

The ERG is generated from electrical currents flowing within the retina in response to photoreceptor stimulation and is most commonly recorded at the corneal surface. Electrical signals detectable at the cornea result from a flow of current within the retina from a source to a sink. The corneal ERG waveform is complex, and its shape is the result of summation of different electrical signals that originate from different retinal structures. The seminal studies of Granit, using increasing depth of anesthesia in cats, identified three “processes” in the combined ERG waveform.^2^ The first process (PI) is a slow positive component now known to originate primarily from the retinal pigment epithelium and typically demonstrated using a direct current (DC) recording method.^3^ PI underlies the ERG c‐wave. The other processes, PII which is a positive waveform, and the faster negative PIII, can be recorded by alternating current (AC) recording, which is the most commonly utilized ERG recording method used today. The PII process primarily originates in bipolar cells and superimposed on the PIII response shapes the b‐wave while the PIII is from current generated in photoreceptors and is a major contributor to the a‐wave. There are contributions to the ERG from other retinal cells including amacrine and ganglion cells and Müller glia. The latter is the source of a slow negative waveform called the slow PIII which is generated by the changes in potassium ion concentrations affecting flow into Müller cells through inward rectifying potassium channels (Kir4.1).^4^, ^5^ In addition to the a‐, b‐, and c‐waves there are several other waveforms recognized. These include the scotopic threshold response (STR), which is predominately a negative response but maybe preceded by a small positive component, that can be recorded in the dark‐adapted eye using very weak stimuli near visual threshold.^6^ Oscillatory potentials are higher frequency waveforms superimposed on the b‐wave. The STR and OPs originate in the inner retina.^7^, ^8^

In the light‐adapted eye, the ERG waveform is driven by cone responses and the b‐wave is shaped by contributions originating from the ON and OFF pathways.^9^ A post‐b‐wave negativity, the photopic negative response (PhNR), is recognized and studies suggest that it originates from ganglion cells.^10^, ^11^ Other waveform components can be present depending on the recording conditions (see Webvision for a thorough review of the ERG http://webvision.med.utah.edu).

The shape and amplitude of the ERG depends on a multitude of factors including the stimulus used, recording conditions, and the physiological state of the retina. The stimulating light can be varied in color, strength, and duration (from the typical brief flash most commonly used, to a longer light exposure for the On–Off ERG). The stimulus used can be altered depending on which class of photoreceptor is being targeted or retinal pathway being investigated. Flickering stimuli are also commonly used to isolate responses driven by cones, which can respond to higher frequency of flashes than rods. The physiological state of the retina influences the ERG waveform, for example, the degree of dark or light adaptation and stimulus strength can be selected to specifically record rod‐driven response, mixed rod, and cone responses and cone‐only responses. Use of a longer duration stimulus to record the On–Off ERG can separate the components that are superimposed in response to a short duration flash.^12^ These include separation of contributions from the OFF pathway from those originating in the ON‐pathway. Waveforms are present at the onset of the light stimulus (the On‐response) and to the offset of light (the Off response—primarily the d‐wave).

The purpose of this paper is to report an inherited ERG abnormality first detected in the Papillon breed of dog. The ERG changes suggest the presence of an underlying negative waveform that is most obvious in the photopic (cone‐driven) ERG as a reduced b‐wave and exaggerated PhNR but also underlies the dark‐adapted ERG, resulting in a significantly larger negative STR and a decrease in the b:a‐wave ratio compared with normal control dogs.

## 2 MATERIALS AND METHODS

### 2.1 Ethics statement

All procedures were performed in accordance with the ARVO statement for the Use of Animals in Ophthalmic and Vision Research and approved by the Michigan State University Institutional Animal Care and Use Committee.

### 2.2 Animals

**FIGURE 1:**
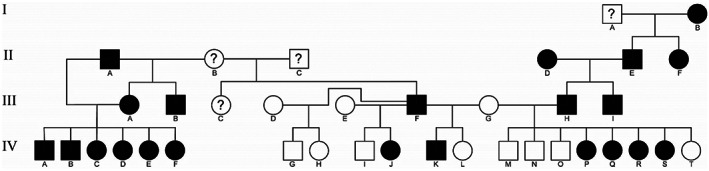
Four generation pedigree of NRE colony dogs. Dogs marked with a “?” are purebred Papillons in which we do not know the phenotype. A dominant mode of inheritance is suggested from the breedings in which two affected male Papillons (III‐F and III‐H) were bred to three different female beagles (III‐D, III‐E, III‐G) and between the four litters produced six puppies had the NRE phenotype (IV‐J, K, P, Q, R and S) and eight puppies had normal ERGs

The dogs used in this study were maintained in a colony at Michigan State University for studies of progressive retinal atrophy in the Papillon. They were housed under 12 h:12 h light:dark cycles and fed a commercial dry food diet. The dogs in this study were either purebred Papillons or Papillons crossed with laboratory beagles. A difference in light‐adapted ERG waveform shape was first identified in PRA‐unaffected dogs in the colony, with some dogs having an unusually exaggerated post‐b‐wave negativity (photopic negative response, or PhNR) the criteria to distinguish between affected and unaffected dogs is described below. We named this ERG phenotype Negative Response ERG (NRE). Further breeding was performed to study this trait (Figure 1). Control dogs were mostly Papillon or Papillon–Beagle crosses that were considered to have a normal photopic ERG waveform. To investigate a possible mode of inheritance, two affected male Papillon dogs were outcrossed with three phenotypically normal female beagles and the resulting puppies were monitored to see if they developed the abnormal waveform. A partial pedigree for the colony showing NRE‐affected dogs is shown in Figure 1.

Electroretinographic assessment of dogs was performed between 1 month and 8 years of age and the condition identified in 20 dogs (10 males and 10 females). Of these, 14 dogs with the ERG abnormality were assessed as puppies between on and 6 months of age, and 10 of these dogs were assessed as adults ≥12 months of age. These were compared with 18 unaffected breed‐ and age‐matched control dogs (nine males and nine females). Of these, 11 were assessed as puppies between one and 6 months of age, and seven dogs assessed as adults ≥12 months of age. Three dogs were followed up to over 8 years of age.

### 2.3 Ophthalmic examination

Routine ophthalmic examinations (including slit‐lamp biomicroscopy and indirect ophthalmoscopy) were performed.

### 2.4 Anesthesia for ERG and imaging

Adult dogs were premedicated with intramuscular acepromazine (0.03 mg/kg acepromazine, Henry Shein Animal Health,), anesthesia‐induced with intravenous propofol (4–6 mg/kg ProposFlo, Abbott Animal Health) and following intubation, maintained with isoflurane (IsoFlo, Abbott Laboratories,) [between 2–3.5% in a 1–2 L/min oxygen flow via a rebreathing circle system for dog over 10 kg and via a Bain system for dog under 10 kg]. Young puppies were induced by isoflurane administered by mask, then intubated and anesthesia maintained with isoflurane.

### 2.5 Electroretinography (ERG)

General procedures for ERGs have been described previously.^13^ Differences in apparatuses and protocols are noted below. Briefly, dogs were dark‐adapted for 1 h and pupils dilated with tropicamide (Tropicamide Ophthalmic Solution UPS 1%, Falcon Pharmaceuticals Ltd.,). The active electrode was a gold‐ringed electrode contact lens (ERG‐Jet electrode, Fabrinal Eye Care, La Chaux‐De‐Fonds, CH). For reference and grounding, platinum needle skin electrodes (Grass Technologies,) were used placed 5 mm lateral to the lateral canthus and over the occiput, respectively. ERGs were recorded using an Espion E^2^ Electrophysiology system with ColorDome Ganzfeld (Diagnosys LLC,).

#### 2.5.1 Dark‐adapted luminance: response series

This consisted of 14 stimulus strengths of white light from below rod b‐wave threshold (starting at −3.7 log cd.s/m^2^) to a flash of 2.82 log cd.s/m^2^. The number of tracings averaged was dependent on the signal‐to‐noise ratio and varied from 20 tracings for the low luminance stimuli to two tracings for the strongest stimuli. Inter‐flash intervals ranged from 2 s for the dim flashes up to 720 s for the brighter flashes. The STR amplitude was measured from the baseline to the negative trough of the waveform. The a‐wave was measured from the baseline to the trough of the a‐wave taking care not to confuse the residual STR seen prior to the b‐wave threshold in response to weaker flashes. The b‐wave was measured from baseline, or when present a‐wave trough, to the peak of the b‐wave allowing for the intrusion of the oscillatory potentials on the underlying b‐wave.

#### 2.5.2 Light‐adapted luminance:response series

The dogs were exposed to a rod‐suppressing background light of 30 cd/m^2^ for 10 min and light‐adapted responses were recorded at four different flash intensities ranging from −0.80–1.4 log cd.s/m^2^ presented on a background white light of 30 cd/m^2^. Finally, cone flicker responses to a flash intensity of 0.39 log cd.s/m^2^ at 33 Hz in the presence of the rod‐suppressing background light were recorded. The post‐b‐wave negativity PhNR was measured from the pre‐flash baseline to the trough of the post‐b‐wave negativity.

#### 2.5.3 On–Off response ERG

ERGs were recorded from long flashes of light (250 mSec duration) with a stimulus of 180 cd/m^2^ presented on a background light of 42 cd/m^2.^ ^14^

### 2.6 Vision testing

Objective assessment of visual ability was performed using a previously described 4‐choice vision‐testing device.^15^, ^16^ In brief, the dogs were placed in a junction box with 4 exit tunnels, the far end of 3 of which were closed, and one randomly selected tunnel left open. With normal vision dogs choose the open tunnel and exit the device. Outcome measures are tunnel choice and exit time. Dogs were tested at 7 different background lighting levels, covering scotopic to photopic vision (0.057, 0.57, 1, 2.8, 20, 41, 750 lux). The time to exit of 14 trials was averaged at each lighting level for each dog.

### 2.7 Spectral‐domain optical coherence tomography

cSLO and SD‐OCT retinal cross‐sectional images were collected as previously described^17^ using a Spectralis OCT + HRA (Heidelberg Engineering,).

### 2.8 Statistical analysis

When two groups of dogs were compared, the data were assessed for normality (Shapiro–Wilk test) and for equal variance (Brown–Forsythe test). Data that passed both tests were compared using an unpaired Student's *t*‐test. Data set failing either were assessed using a non‐parametric Mann–Whitney test. Analyses were by using SigmaPlot (Systat Sofware,).

When datasets including repeated measures from individual dogs a linear mixed‐effects model was used. All variables were initially assessed for homoscedasticity using the Breusch–Pagan test, and for normality using the Shapiro–Wilk test, prior to calculation of F‐statistic using the F‐test of the linear mixed‐effects model (LME). An LME model was employed utilizing the Statsmodels package in Python to examine statistical significance of ERGs performed in dogs with the abnormal ERG phenotype compared with control dogs, fitting the following equation:

(1) $$Y_{\mathrm{ij}}=\beta_{0}+\beta_{1}X_{\mathrm{ij}}+\gamma_{i}+\varepsilon_{\mathrm{ij}}$$

$Y_{\mathrm{ij}}$ is the $j^{\mathrm{th}}$ measured response for subject i, $X_{\mathrm{ij}}$ is the covariate for this response, $\gamma_{i}$ is the random effects parameter for subject i, and $\varepsilon_{\mathrm{ij}}$ is the error parameter for this response. $\beta_{0}$and $\beta_{1}$ are fixed effect parameters for all subjects, corresponding to intercept and slope, respectively, and are fit according to the restricted maximum likelihood (REML) optimized with the Broyden–Fletcher–Goldfarb–Shanno (BFGS) algorithm.^18^

ANCOVA testing was used to compare statistical significance between the means of different groups when modeled with linear regression:

(2) $$y_{\mathrm{ij}}=\mu +\tau_{i}+B\left(x_{\mathrm{ij}}-\bar{x}\right)+\varepsilon_{\mathrm{ij}}$$

The variables *i* and *j* are the *j*th observation of the *i*th categorical group, the dependent variable *y* is modeled as a function of independent variable *x*, μ and $\bar{x}$ are mean parameters derived from the data, and the fitted variables are the effect parameter τ, slope parameter B and error term ε.

After assessment of linearity of regression, homogeneity of error variances, independence and normality of error terms, and homogeneity of regression slopes, mean group differences were assessed using the F‐test.^19^

## 3 RESULTS

### 3.1 Ophthalmic examination

There were no consistent or clinically significant findings on slit‐lamp examination or indirect ophthalmoscopy in any of the dogs included in this study. Importantly, fundus appearance on indirect ophthalmoscopy appeared normal in all dogs including those followed up to 8.5 years of age.

### 3.2 Electroretinography

#### 3.2.1 Light‐adapted ERG

**FIGURE 2:**
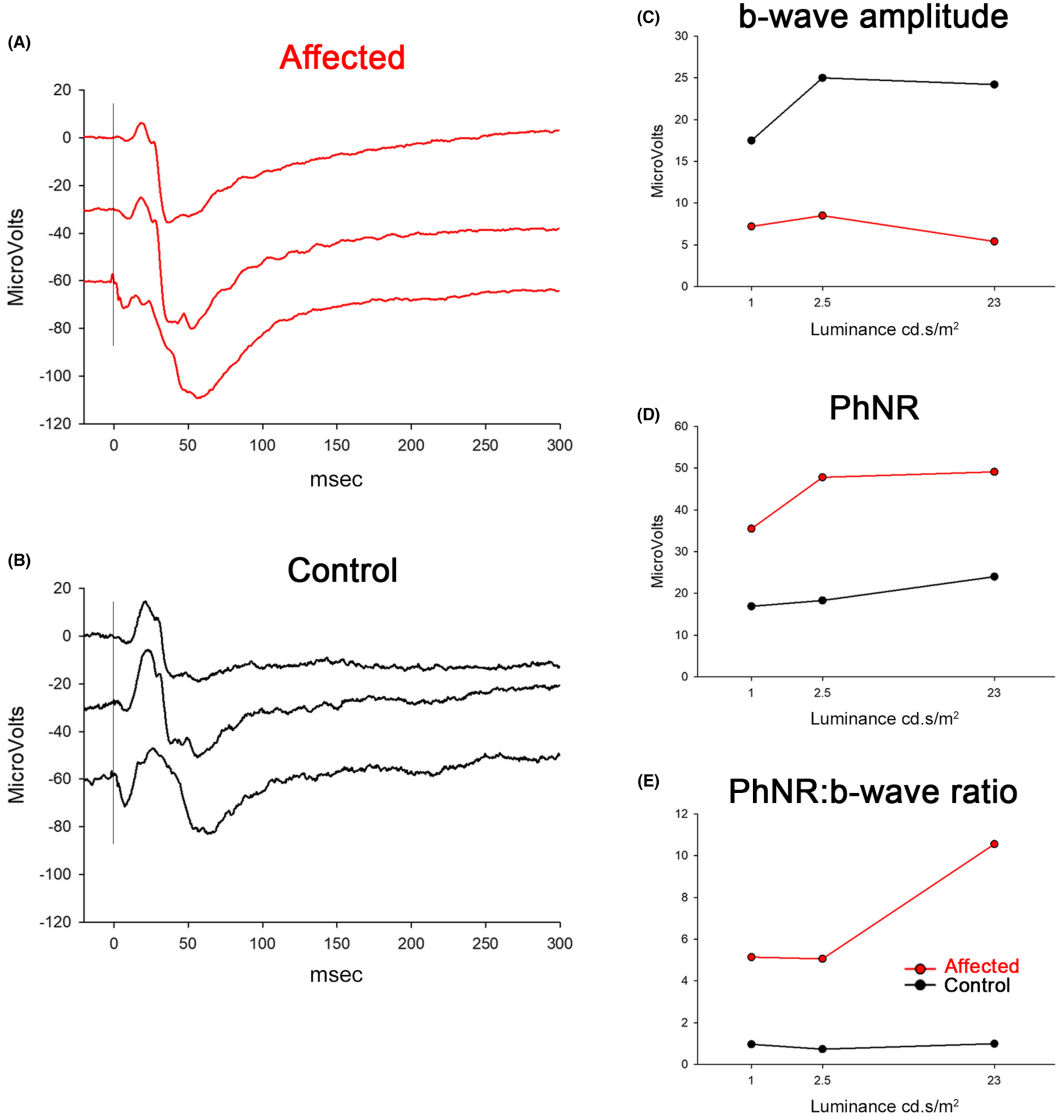
Light‐adapted ERG from a markedly affected (A) and an unaffected (B) dog (dogs II.D—a purebred Papillon and IV.L—a Papillon/Beagle cross, respectively). Note the large PhNR (post‐b‐wave negativity) in the affected dog. In response to the strongest flash (bottom tracing) the b‐wave is almost overwhelmed by the strong negative component. The control dog has normal appearing waveforms showing a photopic hill effect with a decrease in b‐wave amplitude and broadening of the b‐wave with the strongest flash. C, D, and E show the amplitudes of b‐wave, PhNR and the ratio of PhNR to b‐wave for the two dogs. Flash stimuli from top to bottom were 1.0, 2.5 and 23 cd.s/m^2^ on a 30 cd/m^2^ white background light

**FIGURE 3:**
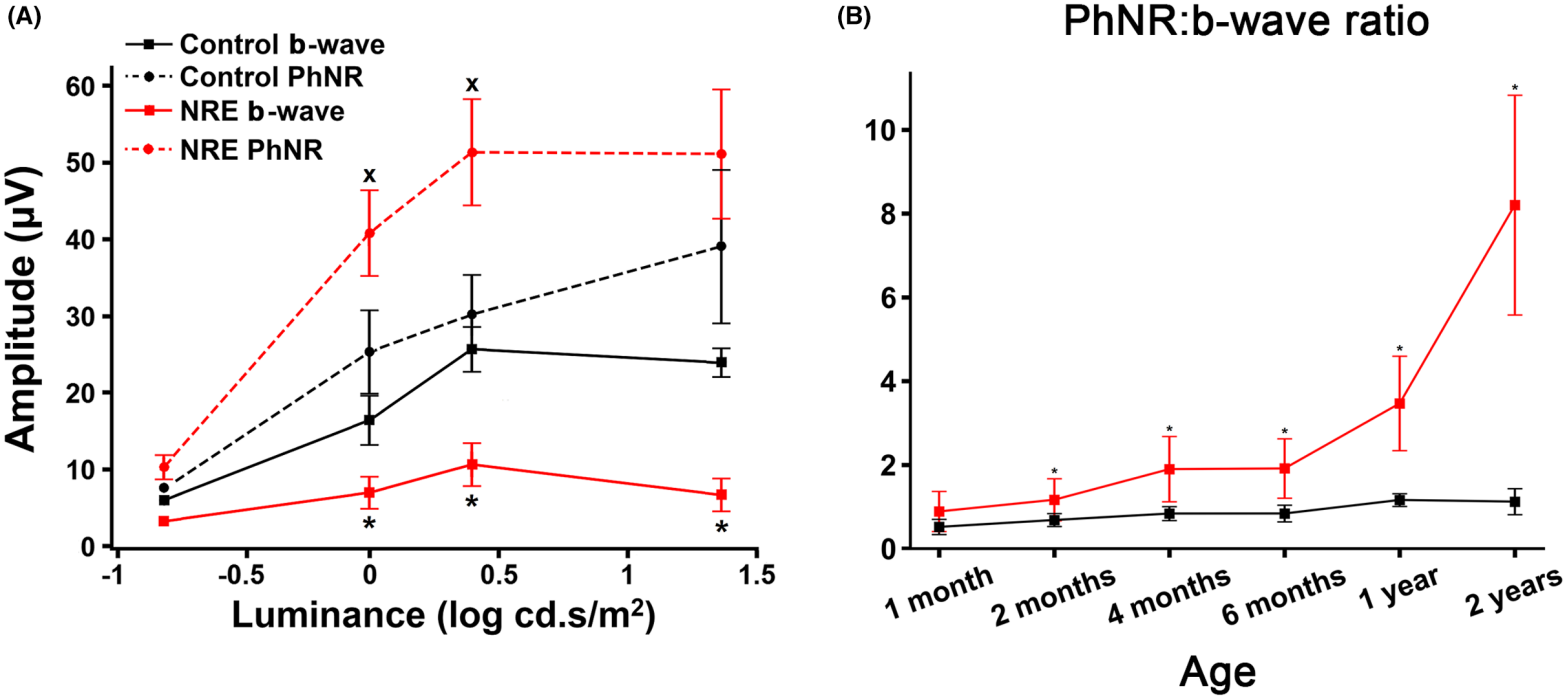
Light‐adapted ERG. A. Mean (±SD) b‐wave and PhNR amplitudes plotted against flash luminance for adult affected (NRE) dogs (red) and unaffected control dogs (black). (x PhNR amplitude difference *p* < .05, * b‐wave amplitude difference *p* < .05). *n* = 13 affected, 5 controls. B. PhNR: b‐wave ratio in response to flash luminance of 0.4 log cd.s/m^2^ plotted against age for affected (red) and unaffected (black) dogs (* *p* < .05). At 1 month *n* = 3 affected, 3 controls; 2 months *n* = 10 affected, 4 controls; at 4 months *n* = 15 affected, 4 controls; at 6 months *n* = 11 affected and 3 controls; at 1 year *n* = 10 affected, 3 controls, at 2 years *n* = 3 affected, 2 controls

We first identified a subset of Papillons with an abnormal shape of the light‐adapted ERG. The waveforms had an exaggerated post‐b‐wave negativity (PhNR) and also a reduced b‐wave. Figure 2A and B show the light‐adapted ERG results from a relatively severely affected Papillon (Figure 2A) compared with an unaffected related dog (Papillon/Beagle cross) (Figure 2B) (dog II.D and IV.L, respectively, on Figure 1). Note that the affected dog has a relatively small b‐wave (Figure 2C) and a large PhNR (Figure 2D), and thus, a larger PhNR:b‐wave ratio compared with the control dog (Figure 2E). The abnormal shape of the ERG of the affected dog became more exaggerated with increased flash strength (Figure 2A). The waveforms have the appearance that there is an underlying negative waveform that is reducing the b‐wave amplitude and exaggerating the PhNR, and that this negative component increased with flash strength. For the purposes of this study, dogs were considered to be NRE‐affected when the light‐adapted PhNR to b‐wave ratio was 1.5 or greater in response to a 0.4 log cd.s/m^2^ flash. The ratio in control dogs with a more typical light‐adapted ERG waveform had a PhNR to b‐wave ratio that was less than 1.5 for this flash stimulus. Figure 3A shows the light‐adapted b‐wave and PhNR amplitude in NRE‐affected dogs over 1 year of age compared with controls with increasing flash stimulus. The PhNR to b‐wave ratio for the 0.4 log cd.s/m^2^ flash is shown in Figure 3B. The mean PhNR:b‐wave ratio was significantly increased in NRE‐affected dogs compared with controls from 2 months of age (Figure 3B). This difference increased with age. For NRE‐affected dogs over 1 year of age, the PhNR:b‐wave ratio was a mean (±SD) of 4.83 (±3.3) compared with that of 1.15 (±0.19) for the unaffected dogs (*p* < .0001) (Table 1).

**TABLE 1:** Average measurement values in NRE and control dogs. The difference in mean values was assessed using the F‐test, and significant *p*‐values (*p* < .05) are given

Measure	Affected	Control	F‐test	*p*‐value
PhNR:b‐wave ratio	4.83 ± 3.30	1.15 ± 0.19	3.92	8.85 × 10^−5^
On–Off PhNR:b‐wave ratio	5.02 ± 2.90	0.26 ± 0.35	3.58	3.34 × 10^−4^
STR amplitude (μV)	46.19 ± 18.42	9.25 ± 4.54	9.92	3.39 × 10^−23^
Scotopic b:a ratio intercept	1.85 ± 0.11	2.34 ± 0.12	2.937	0.003
Scotopic b:a ratio slope	−0.36 ± 0.02	−0.32 ± 0.03	*	*

**FIGURE 4:**
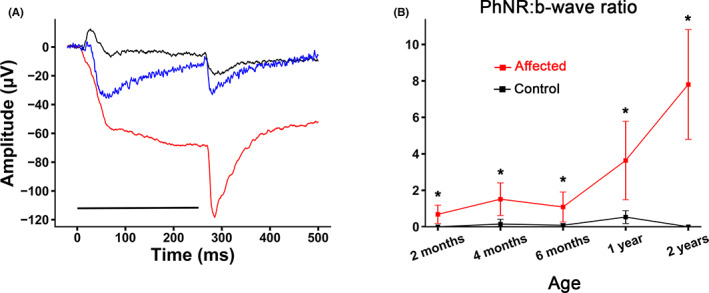
On–Off light‐adapted ERG. A. Dogs with the NRE phenotype had large cornea‐negative deflections during the On‐response (black tracing—unaffected adult—dog IV.L, blue and red tracings different dogs with differing severity of the NRE phenotype, littermates IV.D and IV.F, respectively). The black line under the tracings indicates the duration of the flash. B. Mean (± SD) On‐response PhNR:B‐wave amplitude in control (black) and affected (red) dogs. Note the increase in the ratio of the affected dogs with age. * *p* < .05. Flash stimulus 180 cd/m^2^ presented on a background light of 42 cd/m^2^, 250mSec duration. At 2 months *n* = 6 affected, 10 controls; at 4 months *n* = 12 affected, 8 controls; at 6 months *n* = 11 affected, 6 controls; at 1 year *n* = 10 affected, 5 controls; at 2 years *n* = 3 affected, 2 controls

We also assessed the On–Off ERG by using a longer duration flash (250 mSec). As with the light‐adapted short flash, the b‐wave (On‐response) of affected dogs was markedly reduced and was followed by a large negative component (Figure 4A). In the more extreme instances, the On–Off ERG consisted of a predominantly negative waveform with a small b‐wave superimposed on the down slope (compare the red tracing in Figure 4A with the normal dog tracing [black] and the less severely affected dog [blue tracing]). The ratio of the post‐b‐wave negativity, or On–Off PhNR, to b‐wave was significantly increased in the affected dogs from 2 months of age. With the difference in mean, ratio further increases with age (Figure 4B). For dogs over 1 year of age, the mean PhNR:b‐wave ratio for the On–Off ERG was 5.02 (±2.9) for affected dogs compared with 0.26 (±0.35) for control dogs (*p* < .001) (Table 1).

The 33 Hz light‐adapted cone flicker responses were of normal appearance (data not shown).

#### 3.2.2 Dark‐adapted ERG

**FIGURE 5:**
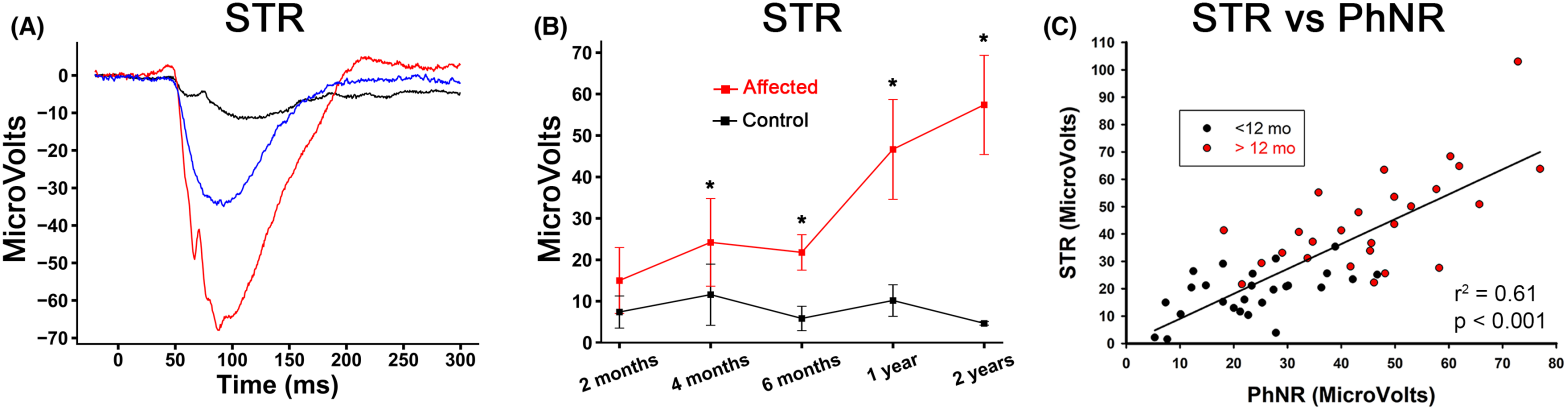
Scotopic threshold response (STR). A. Representative maximum STR recordings from one‐year‐old dogs. Including a normal control dog (black—not shown on pedigree) and two NRE dogs (blue and red, III.G and IV.D). B. The mean (±SD) STR amplitude of control dogs (black) and NRE‐affected dogs (red). Note that mean STR amplitude of NRE dogs increased with age and was significantly greater than that of control dogs. (* *p* < .05). Two months *n* = 6 affected, 4 controls; 4 months *n* = 11 affected, 4 controls; 6 months n = 7 affected, 2 controls; 1 year *n* = 10 affected, 5 controls; 2 years *n* = 3 affected, 2 controls. C. A scatter plot of maximum STR amplitude against the PhNR to a light‐adapted 0.4 log cd.s/m^2^ luminance flash showed a strong correlation. The black symbols are from dogs under 12 months of age (*n* = 26 recording from 12 dogs) and the red symbols for those above 12 months of age (*n* = 26 recordings from 15 dogs)

**FIGURE 6:**
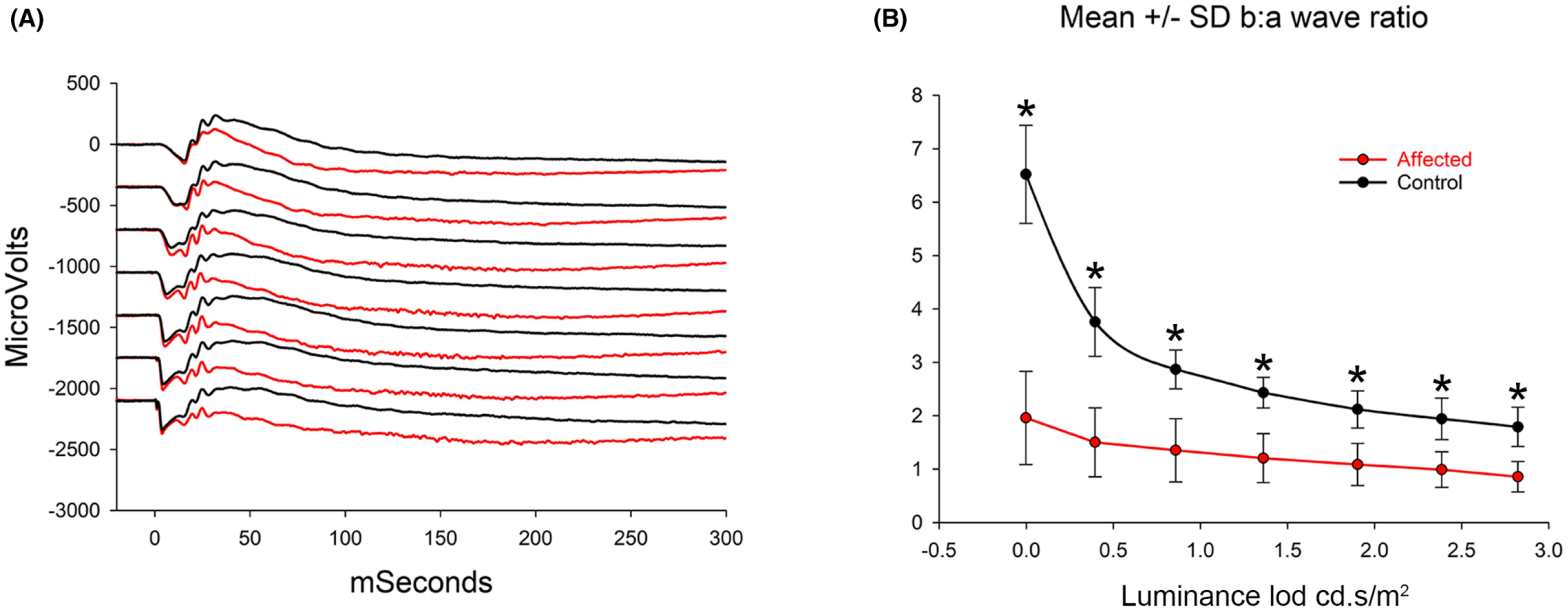
Dark‐adapted ERG luminance series A. Superimposed ERG tracing from an affected (red) and normal control (black) dog (dogs IV.D and IV.L). Note that although the a‐wave amplitudes are very similar the b‐wave of the affected dog is reduced in amplitude. Luminance of the flashes shown: 0, 0.4, 0.9, 1.4, 1.9, 2.3, and 2.8 log cd.s/m^2^. B. The mean (±SD) dark‐adapted b:a‐wave ratios from control (*n* = 10) and NRE (red) dogs (*n* = 5) at 1 year of age (*p < .01 unpaired *t*‐test)

(Figures 5 and 6 **)**. As initially noted for the light‐adapted ERG, closer examination of the dark‐adapted ERG also showed the presence of an underlying negative waveform that was present from the threshold responses up to the mixed rod:cone responses to stronger flashes. The first detected response, the STR, was increased in amplitude in the affected dogs (Figure 5A). The maximal STR amplitude was significantly different from that of control dogs from 4 months of age (Figure 5B) and in the affected dogs the amplitude increased with age while in the control dogs it changed little with age. For NRE dogs over 1 year of age, the mean (±SD) STR amplitude was 46.19 μV (±18.42) while that of controls was 9.25 μV (±4.54) (*p* < .0001 Table 1). There was a strong correlation between STR and short flash PhNR (plotted against each other in Figure 5C). Figure 5D is color coded to see the difference in the scatter plot between NRE dogs under 1 year of age (black symbols) and dogs over 1 year of age (red symbols). This clearly shows the increase in amplitude of both STR and PhNR with age.

When the dark‐adapted luminance:response series was examined (Figure 6), the b‐wave of the NRE dogs was found to be lower than that of the control dogs. Figure 6A shows superimposed dark‐adapted ERGs from an affected and control dog (IV.4 and IV.12, respectively, Figure 1) for the strongest seven flashes. The affected dog has a similar a‐wave amplitudes to the control for most flashes but a noticeably reduced b‐wave. This was most apparent with the stronger flashes in response to which the affected dog had a “negative ERG” (i.e., that in which the b‐wave fails to reach the baseline). To investigate this further, we compared the mean b:a‐wave ratio. This is shown for one‐year‐old dogs in Figure 6B and was significantly smaller in affected compared with controls for each flash luminance following a‐wave threshold.

### 3.3 Vision testing

**FIGURE 7:**
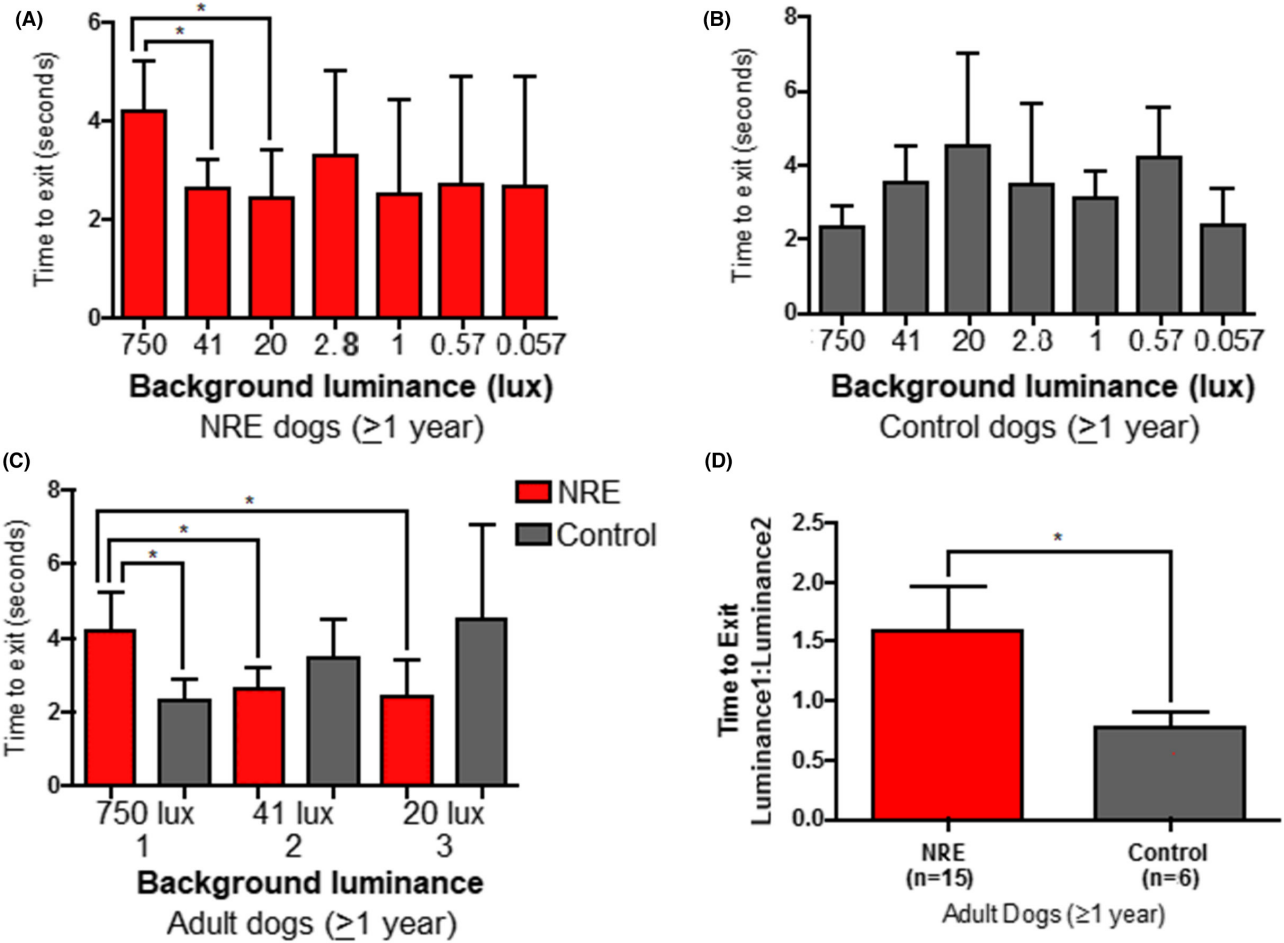
Four‐choice vision testing results in adult dogs. A. NRE‐affected dogs (*n* = 16). B. Control dogs (*n* = 5). C. A comparison between the results at the two highest light levels between NRE dogs (red) and control dogs (Black). Dogs with the NRE phenotype took a significantly longer time to exit at the highest light level than at the next two lower light levels and also when compared to controls at the highest light level. D. The ratio of time to exit for the highest and second highest light levels for the NRE dogs (red) and controls (black). *p < .05

Using a four‐choice vision‐testing device the correct exit choice and time to exit was recorded for a range of lighting levels from photopic to scotopic levels. There was no significant difference in exit choice between the affected dogs and the controls (data not shown). However, the NRE dogs took significantly longer to exit compared with control dogs under the brightest luminance (750 lux) (independent *t*‐test; *p* < .01) (Figure 7A vs B). Furthermore, the NRE dogs were slowed to exit the device at the brightest lighting level compared with the next two lower lighting levels (41 and 20 lux) (Figure 7C) (paired *t*‐test, *p* < .05). The exit time for control dogs was not significantly different between the highest 3 lighting levels (Figure 7B). The longer exit time for the brightest light condition is further demonstrated by looking at the ratio of the exit time at 750 lux to the exit time at 41 lux. The ratio in NRE dogs was significantly greater than in the control group (Figure 7D). There were no significant differences in time to exit between the control and NRE dogs for the 6 dimmest lighting levels (Table 2).

**TABLE 2:** Mean (± SD) time to exit in vision testing of NRE and control dogs

		Light level (lux)						
		750	41	20	2.8	1	0.57	0.057
Time to exit (sec)	Control (*n* = 6)	2.64 ± 1.01	3.54 ± 1.94	4.05 ± 2.53	3.38 ± 1.95	3.21 ± 0.99	4.45 ± 1.39	2.36 ± 1.03
	Affected (*n* = 15)	4.80 ± 1.61	2.60 ± 0.52	3.14 ± 1.33	3.40 ± 2.14	3.23 ± 1.44	3.47 ± 1.74	3.66 ± 1.86

### 3.4 Spectral‐domain optical coherence tomography (SD‐OCT)

**FIGURE 8:**
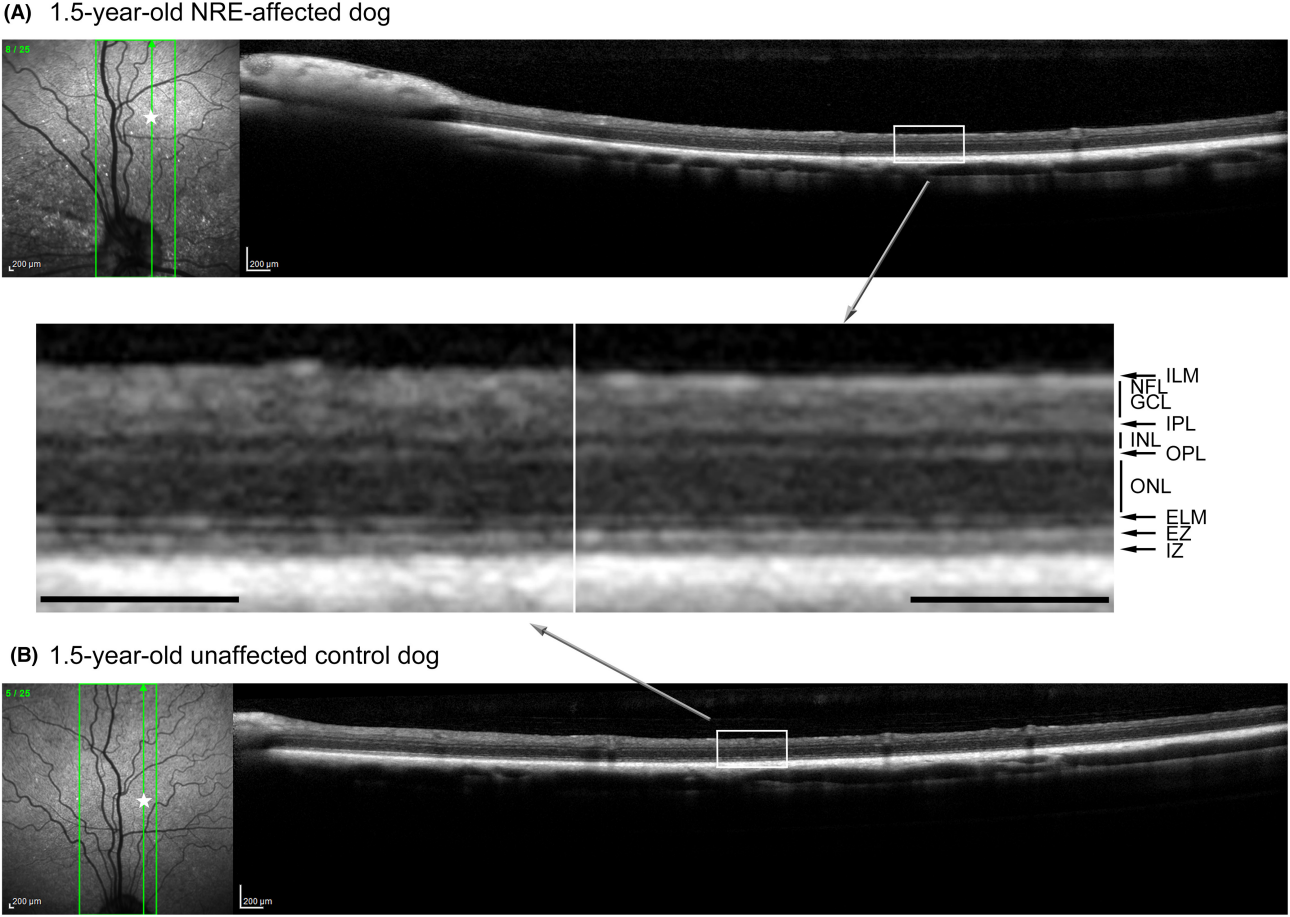
Comparison of SD‐OCT in a 1.5‐year‐old NRE‐affected (III.B) (A) and control dog (not shown on pedigree) (B). Between the two images, magnified regions of the central retina are shown, and retinal layer thickness are very similar. The star on the cSLO infrared image indicate the region of the magnified box. (ELM, external limiting membrane; EZ, ellipsoid zone; GCL, ganglion cell layer; ILM, internal limiting membrane; INL, inner nuclear layer; IPL, inner plexiform layer; IZ, interdigitation zone; NFL, nerve fiber layer; ONL, outer nuclear layer; OPL, outer plexiform layer). Scale = 200 μm

**FIGURE 9:**
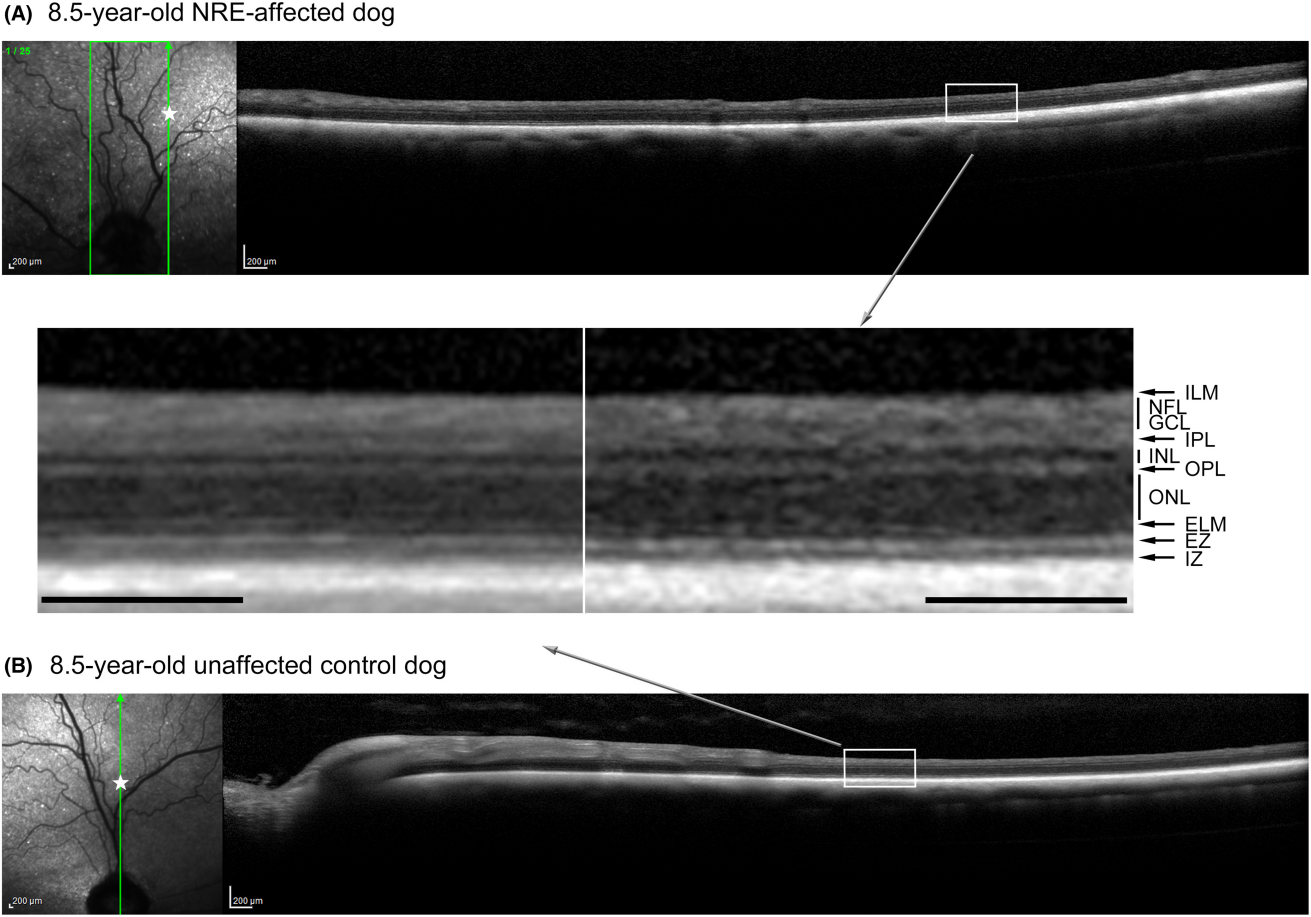
Comparison of SD‐OCT in 8.5‐year‐old NRE‐affected (I.B) (A) and control dog (not shown on pedigree) (B). Between the two images, magnified regions of the central retina are shown, and retinal layer thickness are very similar. The star on the cSLO infrared image indicate the region of the magnified box. (ELM, external limiting membrane; EZ, ellipsoid zone; GCL, ganglion cell layer; ILM, internal limiting membrane; INL, inner nuclear layer; IPL, inner plexiform layer; IZ, interdigitation zone; NFL, nerve fiber layer; ONL, outer nuclear layer; OPL, outer plexiform layer). Scale = 200 μm

SD‐OCT was performed on both younger NRE‐affected and control dogs (Figure 8) as well as a dog at 8 years of age (Figure 9). Both younger and older dogs showed normal retinal lamination and there was no evidence of a retinal degenerative process.

### 3.5 Pedigree analysis

Test matings were performed to cross two affected male Papillons (Figure 1; III‐F and III‐H) with phenotypically normal beagles (III‐D, III‐E, III‐G). A total of four litters were obtained and the puppies investigated by ERG for the NRE phenotype. One of the NRE‐affected males was bred to each of the three beagle females resulting in three litters of two puppies each. In one litter, both were unaffected, whereas the other two litters consisted of one affected and one unaffected. The other NRE‐affected male was bred with one of female beagles resulting in eight puppies, four of which were affected. Thus, from the four breedings, 14 puppies were produced, six of which had the NRE ERG phenotype, and eight were unaffected. This segregation pattern is in keeping with a dominant mode of inheritance for the NRE trait. Both males and females are affected suggesting an autosomal mode of inheritance.

## 4 DISCUSSION

In this study, we identified an ERG phenotype in dogs which we termed a Negative Response ERG (NRE), characterized by an exaggerated negative component to the ERG. Although most obvious in the light‐adapted ERG waveforms as a large PhNR like response, and in the On–Off ERG as an increase negative component to the On‐response (Figure 4), close examination of the dark‐adapted ERG revealed that it was also affected, having increased negative STR amplitudes and a reduced b‐ to a‐wave ratio (Figures 5 and 6). The response to the strongest flashes often resulted in a negative ERG (a‐wave amplitude is greater than b‐wave amplitude such that the b‐wave does not extend above the baseline). The severity of the ERG changes increased with age (Figure S1). Although for some measures significant differences in mean amplitudes or ERG waveform ratios were present as early as two months of age, the differences were more obvious by 6–12 months of age. There was some variation in the severity of the amplitude changes between dogs (see Figures 4A and 5A).

Vision testing of the adult NRE‐affected dogs suggested a mild impairment of visual function in bright light conditions. This was shown as a significantly slower exit time from a four‐choice vision‐testing device at the brightest light level (750 lux).

The changes to the light‐adapted ERG in the NRE‐affected dog appear similar to a negative light‐adapted ERG that is reported to develop in the Royal College of Surgeons (RCS) rat.^20^ The RCS rat has a mutation in the receptor tyrosine kinase (*Mertk*) gene with a resulting phenotype characterized by a failure in photoreceptor outer segment phagocytosis by the retinal pigment epithelium leading to a progressive retinal degeneration.^21^ The light‐adapted ERG of affected rats develops an increased negativity. This negativity was shown to have a different origin to the photopic negative response, which is thought to originate from ganglion cells, with amacrine cell contributions in some species.^10^, ^22^ The RCS rats were shown to have an increase in the number of Kir4.1 channels on Müller cells, potentially allowing for an increased influx of potassium ions into the Müller cells than normal, explaining the larger negative component to the ERG waveform. Potassium influx through Müller cell Kir4.1 channels has been shown to drive the normal slow PIII response.^5^, ^23^ In the RCS rat, the increase in Kir4.1 channels develops as a secondary process in the degenerating retina rather than as a primary result of a loss in Mertk function. The NRE dogs, unlike the RCS rat, have a functional abnormality that does not appear to be associated with retinal degeneration. However, it is conceivable that the functional change in the NRE dogs could also be due to an abnormality of channel characteristics generating a corneal negative ERG component as demonstrated in the RCS rat. Although we termed the light‐adapted ERG change an exaggerated PhNR, it is possible that as with the RCS rat it is not a true PhNR and may have different origins. We need to ascertain the cause of the ERG change to determine if this is the case.

In addition to changes in the light‐adapted flash and On–Off response, there was an exaggerated negative component to the dark‐adapted ERG. This was present from response threshold leading to an larger amplitude STR and a significant reduction in the b‐:a‐wave ratio. The STR is a waveform recorded from weak stimuli that are close to the psychophysical limit of vision. It is post‐receptoral and believed to originate from changes in extracellular potassium levels that affect Müller cells and may have additional contributions from ganglion cells and amacrine cells.^6^, ^7^, ^24^, ^25^ Although there may be a small cornea‐positive component, the dominant waveform in most species is a cornea‐negative deflection.^26^ The increased STR amplitude in NRE dogs had a significant correlation between with the increased PhNR amplitudes. The negative component underlying the dark‐adapted ERG of the NRE dog resulted in the reduced b:a‐wave ratio and was present at all stimuli used. These findings could be in keeping with a mechanism involving abnormal changes in potassium concentration affecting Müller and proximal retinal cells perhaps due to alterations in cation channel function or expression as discussed above.

NRE dogs showed no funduscopic signs of retinal thinning over the period that they were examined, and dogs imaged at 8.5 years of age by SD‐OCT did not have any evidence of retinal degeneration. Together, this suggests that retinal degeneration is not a feature of the NRE phenotype although we cannot rule out changes appearing in older dogs. So, although the ERG abnormalities do become more pronounced over the first year or two of life, they do not seem to result in retinal degeneration. Detailed histological studies would be required to further confirm this. Vision testing suggested a mild impairment of vision in bright lighting conditions. This suggests that the changes that generate the NRE‐associated change in retinal electrical activity may also compromise cone pathways under stronger lighting conditions.

Negative ERG waveforms can result from conditions resulting in a lack of either transmission from photoreceptors to bipolar cells, or a lack of ON‐bipolar cell function, such as seen in many forms of congenital stationary night blindness (CSNB). Human patients, mouse models, dogs, and horses with gene mutations affecting the ON‐pathway responses with resultant CSNB have a negative shape waveform for the dark‐adapted ERG with a reduction or lack of the b‐wave.^27^, ^28^, ^29^, ^30^ The ERG changes in these conditions are due to a lack of generation of the b‐wave rather than superimposition of a negative waveform such as an increase in the slow PIII response that we theorize underlies the NRE phenotype.

Human patients with gene mutations associated with congenital glycosylation defects have ERG abnormalities suggestive of ON‐pathway defects and have changes to the On‐response of the On–Off ERG somewhat similar to the changes we have identified in the NRE dogs.^31^ Such patients have a childhood multisystem disorder as well as a progressive retinal degeneration.^32^ Sieving also identified human patients with a negative light‐adapted ERG waveform which he described as a hyperpolarizing pattern cone ERG.^14^ The recorded ERGs were somewhat similar to the NRE ERG waveform reported here. He further postulated that the affected patients with this ERG phenotype had dysfunction of the cone ON‐pathway. To the authors' knowledge, the cause of the hyperpolarizing pattern cone ERG in human patients has not been reported.

In summary, we have described a hereditary retinal functional abnormality with a very distinct ERG phenotype in the dog. The ERG changes appear to result from an increase in a negative waveform that is present in response to rod stimuli from those close to response threshold in the dark‐adapted eye as well as to stronger flashes in light‐adapted eyes. This negative waveform appears to increase with increasing stimuli. Test breeding and pedigree analysis suggest an autosomal dominant mode of inheritance. Further genetic studies are underway to try and further understand the condition.

## AUTHOR CONTRIBUTIONS

SMP‐J conceived the project, analyzed data, and wrote the manuscript. NP analyzed data, measured ERGs, performed statistical analysis, and was involved in writing the manuscript. LMO analyzed data, performed SD‐OCT, and edited the manuscript. KJG analyzed ERG data and performed vision testing. FMM was involved in analysis of data. JQ was involved in colony care and assessment and performed ERGs. PAW was involved in colony management, pedigree analysis, data analysis, and manuscript editing.

## CONFLICTS OF INTERESTS

The authors have no conflict of interest.

## Supporting information


Figure S1
