# Supplementary material for: An unusual inherited electroretinogram feature with an exaggerated negative component in dogs
Source: Vet Ophthalmol. 2022 Jun 17;25(5):385–97. doi: 10.1111/vop.12998 (PMC9540982; doi:10.1111/vop.12998)
Supplement: Supplementary file 1 — Figure S1 [file VOP-25-385-s001.pdf]

SUPPORTING INFORMATION

An unusual inherited electroretinogram feature with an exaggerated negative component in dogs

[redacted]

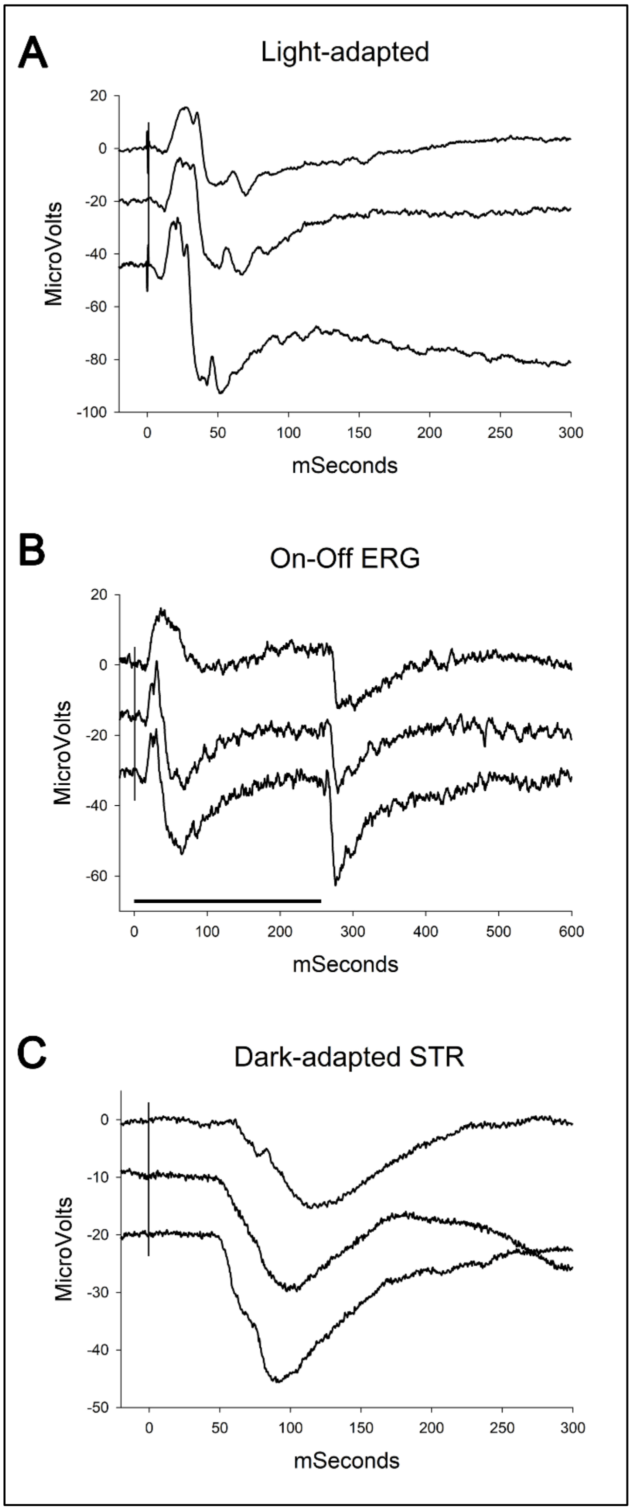

Supp Figure 1. Changes with age in a representative NRE-affected dog. Representative tracings from a dog with the NRE phenotype are shown at 2, 6, and 12 months of age for the light-adapted (A), On-Off ERG (B), and STR (C) (Dog IV.F). The post b-wave negativity and STR increase with age.
